# A Flavonoid Compound Promotes Neuronal Differentiation of Embryonic Stem Cells *via* PPAR-β Modulating Mitochondrial Energy Metabolism

**DOI:** 10.1371/journal.pone.0157747

**Published:** 2016-06-17

**Authors:** Yu-qin Mei, Zong-fu Pan, Wen-teng Chen, Min-hua Xu, Dan-yan Zhu, Yong-ping Yu, Yi-jia Lou

**Affiliations:** 1 Institute of Pharmacology and Toxicology, College of Pharmaceutical Sciences, Zhejiang University, Hangzhou, Zhejiang, People’s Republic of China; 2 Institute of Materia Medica, College of Pharmaceutical Sciences, Zhejiang University, Hangzhou, Zhejiang, People’s Republic of China; Temple University School of Medicine, UNITED STATES

## Abstract

Relatively little is known regarding mitochondrial metabolism in neuronal differentiation of embryonic stem (ES) cells. By using a small molecule, present research has investigated the pattern of cellular energy metabolism in neural progenitor cells derived from mouse ES cells. Flavonoid compound **4a** faithfully facilitated ES cells to differentiate into neurons morphologically and functionally. The expression and localization of peroxisome proliferator-activated receptors (PPARs) were examined in neural progenitor cells. PPAR-β expression showed robust upregulation compared to solvent control. Treatment with PPAR-β agonist L165041 alone or together with compound **4a** significantly promoted neuronal differentiation, while antagonist GSK0660 blocked the neurogenesis-promoting effect of compound **4a**. Consistently, knockdown of PPAR-β in ES cells abolished compound **4a**-induced neuronal differentiation. Interestingly, we found that mitochondrial fusion protein Mfn2 was also abolished by sh*-PPAR-β*, resulting in abnormal mitochondrial Ca^2+^ ([Ca^2+^]_M_) transients as well as impaired mitochondrial bioenergetics. In conclusion, we demonstrated that by modulating mitochondrial energy metabolism through Mfn2 and mitochondrial Ca^2+^, PPAR-β took an important role in neuronal differentiation induced by flavonoid compound **4a**.

## Introduction

Stem cell differentiation is associated with changes in metabolism and function. Understanding these changes during neuronal differentiation is important in the context of stem cell research, neurodegenerative diseases and regenerative medicine. While much has been learned about the molecular events involved in neuronal differentiation [1], relatively little is known regarding their bioenergetic demands and how closely their energy metabolism is governed by the genetic developmental programme [2].

Mitochondria are vital ATP-generating cellular organelles, and they are particularly essential in the nervous system [3]. The increased mitochondrial activity associated with differentiation provides ATP to support fundamental energy-demanding processes involved in neuronal differentiation [1]. Mitochondria dynamics are essential for neuronal functions since they regulate mitochondrial number, location, morphology and function [1]. Mitochondrial fission and fusion are regulated by two distinct protein complexes. Drp (dynamin-related protein) and Fis1, mediate mitochondrial fission, whereas mitofusins (Mfn1 and Mfn2) and OPA1 (Optic Atrophy-1) regulate mitochondrial fusion [1, 3, 4]. In mammals, mutations in Mfn2 cause Charcot-Marie-Tooth (CMT) type 2A, a peripheral neuropathy characterized by axonal degeneration [5]. Cerebellum-specific Mfn2-knockout mice revealed the importance of mitochondrial fusion in protecting cerebellar neurodegeneration [6]. These findings suggested a key role of Mfn2 in nervous system. Furthermore, recent reports demonstrated a new function of Mfn2, which was tethering the endoplasmic reticulum and mitochondria to control the efficiency of mitochondrial uptake of Ca^2+^ ions [7–9]. However, relatively little is known about the potential role of Mfn2 and its upstream determinants in cellular energy metabolism of neuronal differentiation.

Mitochondrial biogenesis and dynamics require coordinated changes in the metabolic enzymes of oxidative phosphorylation and fatty acid oxidation [10]. Peroxisome proliferator-activated receptors (PPARs) are lipid-activated transcription factors belonging to the nuclear receptor superfamily [10]. Three PPAR isotypes (α, β, and γ) have been distinguished by tissue- and developmental-specific expression patterns [11]. PPAR-α, which is mainly rich in tissues that have high-energy demands, was involved in cardiomyocyte differentiation of mouse ES cells *in vitro* [12]. PPAR-γ is found primarily in the adipose tissue and plays an important role in adipose differentiation [13]. PPAR-β is the most ubiquitously expressed with a controversial role [10, 11]. The important role of lipid molecules in brain development is well known [14]. All three PPAR isotypes are expressed in the brain, while PPAR-β is the most abundant subtype [15]. Recent findings demonstrated that modulation of PPAR-β expression might be an important part of brain pathology [16]. The presence and possible modulation of these receptors were also examined in embryonic rat cortical neurons during their *in vitro* maturation [14]. The results suggested a potential role of PPAR-β in neuronal maturation. In addition, a neuronal differentiating effect of PPAR-β was demonstrated in human neuroblastoma cell line SH-SY5Y [17, 18]. Moreover, it was reported that retinoic acid (RA) induced neurogenesis by activating both retinoic acid receptors (RARs) and PPAR-β in P19 mouse embryonal carcinoma cell line [19]. However, the PPAR isotype expressions and their downstream effects during neuronal differentiation of ES cells have not been investigated so far.

The role of small molecules in stem cell biology is emerging [20]. Such molecules will likely provide new insights into mitochondrial metabolism in neuronal differentiation of ES cells, and may ultimately contribute to effective medicine for tissue repair and regeneration [21]. Our previous work showed that some natural flavonoid compounds, icaritin (ICT) [22] and isobavachin (IBA) [23] had significant neurogenesis-inducing activities. In the present study, we used a newly-screened flavonoid compound **4a** as a probe of underlying biology, and aimed to elucidate PPARs expressions and several elements of cellular energy metabolism in neuronal differentiation of mouse ES cells.

## Results

### Flavonoid compound 4a promoted neuronal differentiation of mouse ES cells

**Fig 1:**
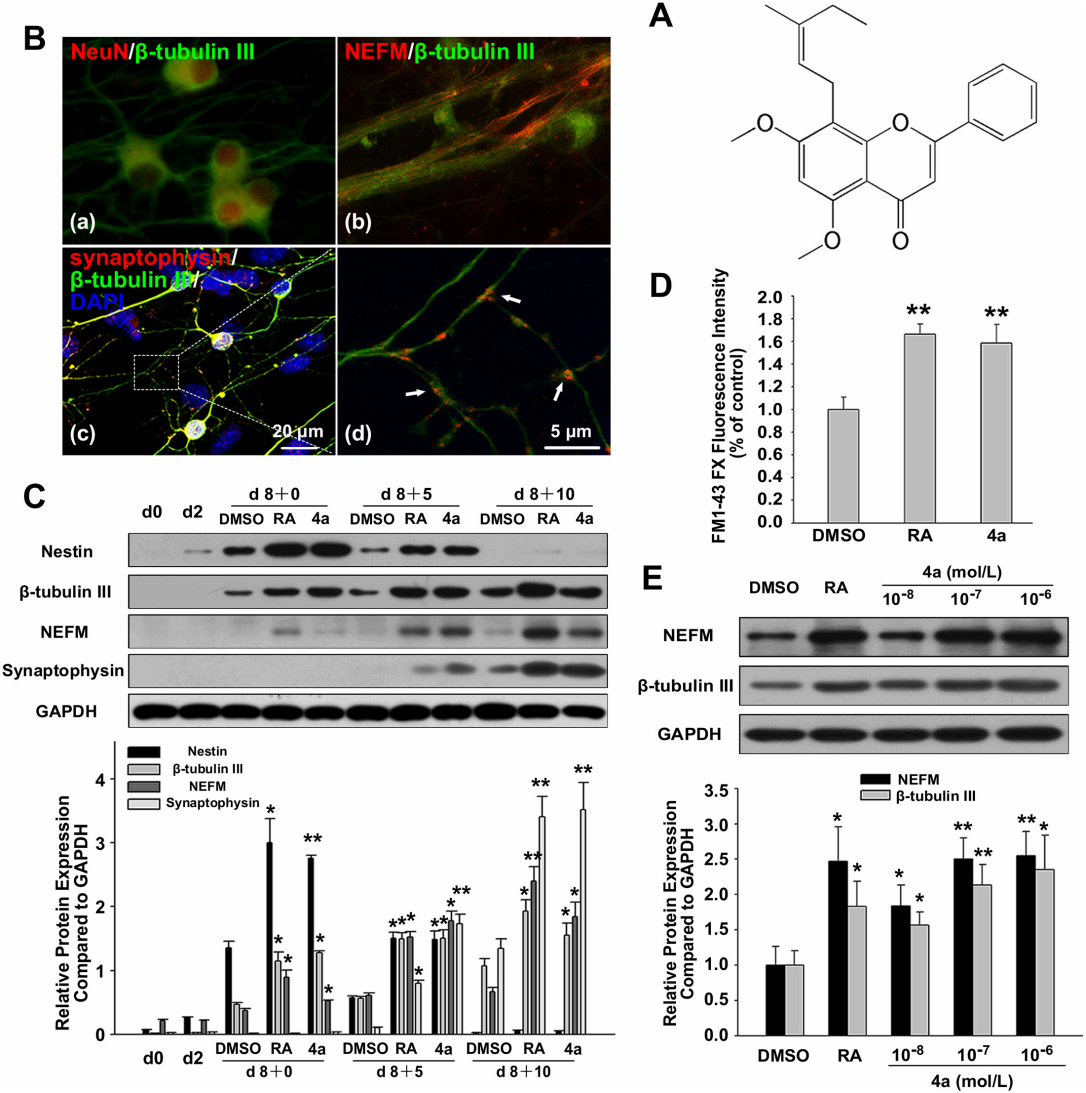
Flavonoid compound 4a promoted neuronal differentiation of mouse ES cells. **A:** Structure of compound **4a. B:** (a-c) Double Immunofluoresence staining for neural specific markers in ES-derived neurons induced by **4a** on d 8+10. (d) The arrows indicated the areas of synaptophysin/β-tubulin III colocalization. Nuclei were stained with DAPI. Bar = 20 μm (a-c), and 5 μm (d). **C:** Developmental-dependent neural specific proteins expression induced by **4a** (10^−7^ mol/L) during the differentiation course. **D:** Compound **4a** promoted depolarization-induced synaptic vesicle recycling function of ES-derived neurons. The neuronal property of synaptic vesicle recycling was detected by FM 1-43FX. The data was obtained from the index of fluorescence intensity normalized to MTT absorbance. **E:** Dose-dependent neural specific proteins expression in ES-derived neurons induced by **4a** on d 8+10. Data were represented as the mean ± S.D. of three independent experiments. Statistical significance was set as *P<0.05, **P<0.01 vs. DMSO control. Retinoic acid (RA) was used as a positive control.

Compound **4a** (5,7-dimethoxy-8-(3-methyl-pent-2-enyl)-2-phenyl-chromen-4-one) was offered in this case by Prof. Dr. Yong-ping Yu, which were synthesized by previous methods [24]. The structure of compound **4a** was shown in Fig 1A. To induce neuronal differentiation, a typical 4−/4+ protocol was used (S1 Fig). After compound **4a** treatment, the expression and localization of neuron-specific proteins were evaluated by immunocytochemistry. Among them, β-tubulin III and neuronal nuclei (NeuN) [25] were neuron cytoplasm and nucleus house-keeping marker, neurofilament 160 (NEFM) [26] was axons marker, and synaptophysin [27] was synaptic vesicles marker. The results in Fig 1B showed that compound **4a** could induce neuron-specific proteins expression. In consistent with this, western blot analysis showed compound **4a** could upregulate the neural specific proteins expression in a developmental way, providing the fundamentals for synaptic vesicle recycling (Fig 1C). Nestin is a neural progenitor marker, which expressed at early differentiation stage. Compound **4a** induced Nestin expression robustly on day 8 of differentiation (Fig 1C), indicating that its neurogenesis-inducing effect appeared as early as neural progenitor cells formation period. The neuronal property of synaptic vesicle recycling was detected by FM 1-43FX. The dye can be internalized from the culture medium during synaptic vesicle recycling, in response to a high concentration of potassium ions in the medium [28]. As a result, cells that possess the neurogenic function display increased FM1-43FX fluorescence. The fluorescence intensity in ES-derived neurons induced by **4a** was similar to that of cells treated with retinoic acid (RA) (Fig 1D). Since synaptic vesicle recycling is a neuron-specific function, we confirmed compound **4a** could induce functional neuronal differentiation. Semiquantitative analysis indicated that the neurogenesis-inducing effect of compound **4a** was in a dose-dependent manner at the terminal differentiation point (Fig 1E).

### PPAR-β was involved in compound 4a-induced neuronal differentiation of ES cells

**Fig 2:**
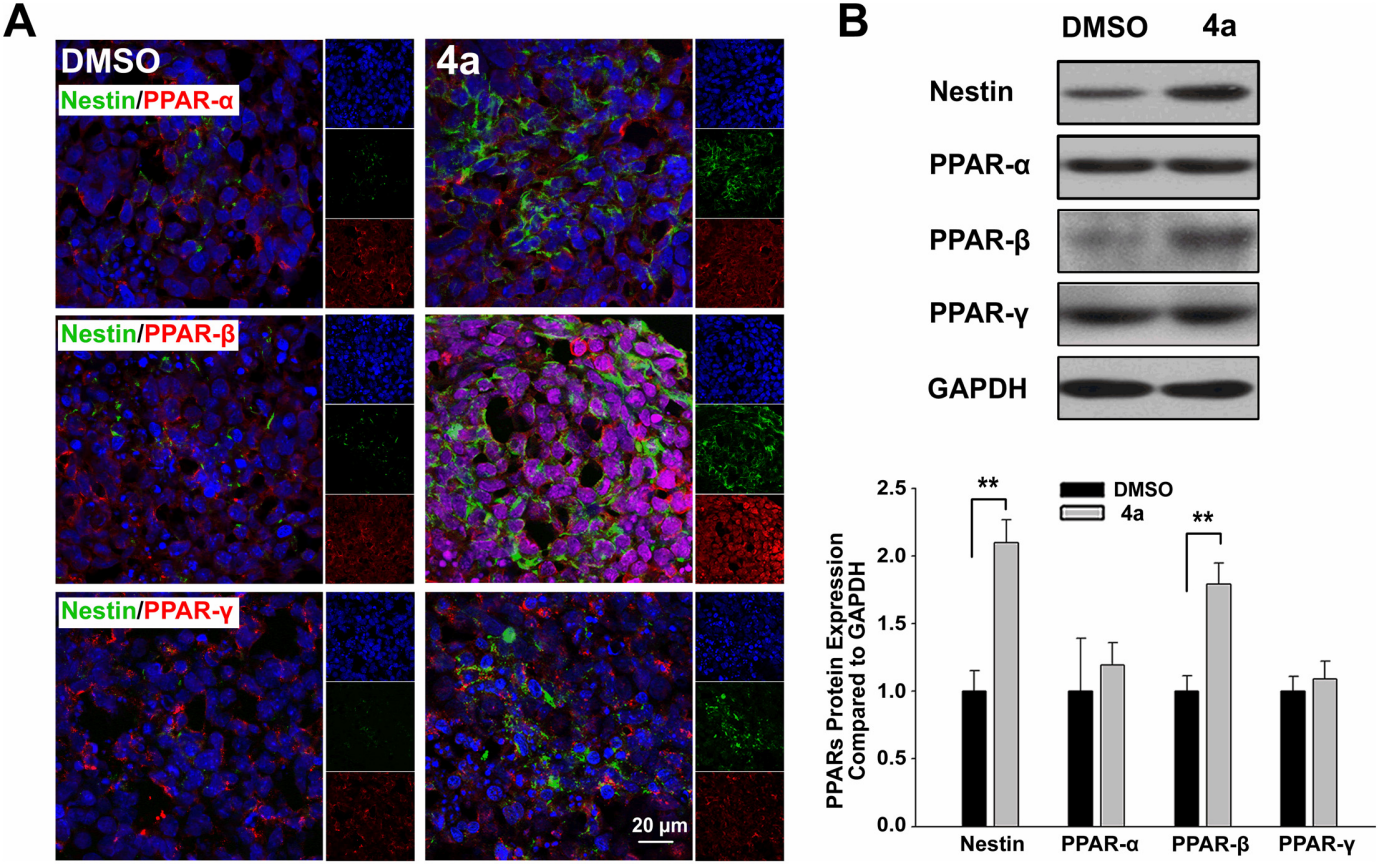
PPAR-β was involved in neuronal differentiation of ES cells at early stage. **A:** Double immunostaining for PPARs and Nestin in ES-derived neural progenitor cells. After treatment with compound **4a**, EBs (d 8) was prepared for frozen section. The expression and localization of PPARs and Nestin were examined. Nuclei were stained with DAPI, and DMSO was set as solvent control. **B:** PPARs protein expression in ES-derived neural progenitor cells. Data were represented as the mean ± S.D. of three independent experiments. Statistical significance was set as **P<0.01 vs. DMSO control.

A developmental-dependent expression of PPAR isotypes were identified by western blot analysis (S2 Fig). Data showed that after compound **4a** treatment, PPAR-α deceased during the differentiation, while PPAR-β gradually increased both in the early (d 8) and late (d 8+10) stage. PPAR-γ, however, did not change during neuronal differentiation. Therefore, we focused on the early stage of neuronal differentiation in the following research. Expression and distribution of PPARs were revealed by double staining PPAR isotypes with neural progenitor marker Nestin on d 8. The percentages of Nestin positive cells were counted in five different EBs frozen sections respectively, and the result was about 22.2±5.9% in DMSO group and 67.8±3.8% in **4a** group. Fig 2A showed that all PPARs were expressed in ES-derived neural progenitors. Typically, PPAR-β was mainly found in cell nuclei with higher fluorescence intensity. The increased expression of PPAR-β was also identified by western blot analysis, while both PPAR-α and PPAR-γ stayed the same (Fig 2B), suggesting a key role of PPAR-β in early neuronal differentiation of ES cells induced by compound **4a**.

**Fig 3:**
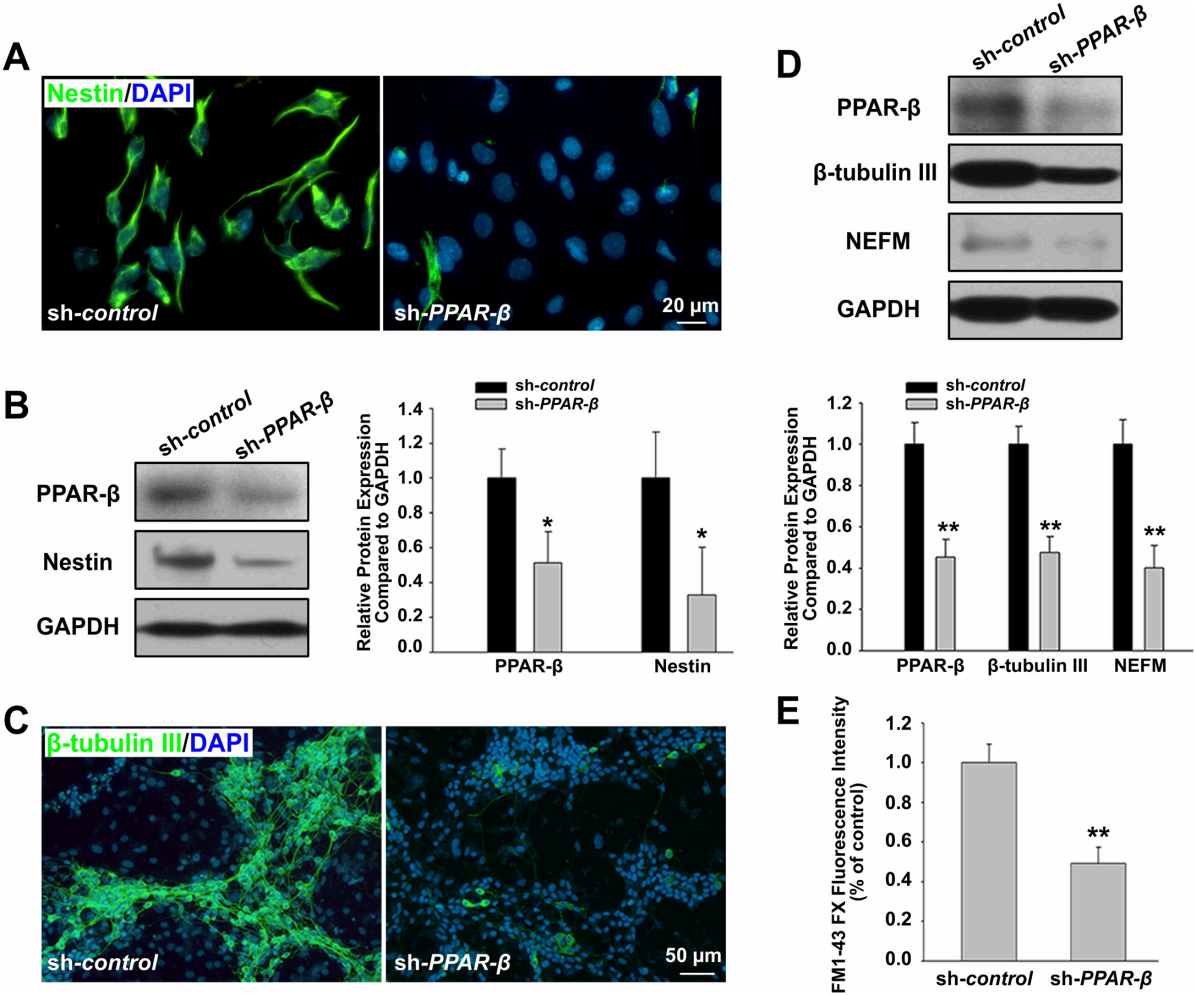
Effects of *PPAR-β* gene silencing on 4a-induced neuronal differentiation of ES cells. ES cells were treated with sh-*PPAR-β* plasmid for 24 h, and then cultured according to 4-/4+ protocol. **A-B:** Nestin expression was detected by immunostaining and western blot assay on d 8. Nuclei were stained with DAPI. Bar = 20 μm. **C:** β-tubulin III expression was detected by immunostaining on d 8+10. Nuclei were stained with DAPI. Bar = 50 μm. **D:** β-tubulin III and NEFM expression was detected by western blot assay on d 8+10. The data was obtained from the index of each proteins compared with GAPDH. **E:** The neuronal property of synaptic vesicle recycling was detected by FM1-43FX. The data was obtained from the index of fluorescence intensity normalized to MTT absorbance. Statistical significance was set as *P<0.05, **P<0.01 vs. sh-*control* group. Data represent mean ± S.D. of three independent experiments.

The Effects of PPAR-β agonist L165041 and antagonist GSK0660 on compound **4a**-induced neuronal differentiation were then evaluated (S3 Fig). Treatment with PPAR-β agonist L165041 alone or together with compound **4a** significantly promoted neuronal differentiation, while antagonist GSK0660 blocked the neurogenesis-promoting effect of compound **4a**. We further investigated whether PPAR-β knockdown in ES cells could block compound **4a**-induced neurogenesis of ES cells. Transfection and silencing efficiency of sh*-PPAR-β* plasmid was evaluated before the experiment (S4 Fig). Viability and pluripotency of ES cells were not affected by sh*-PPAR-β*. Consistently, knockdown of PPAR-β abolished compound **4a**-induced neuronal differentiation. The results showed that Nestin was downregulated in neural progenitor cells (Fig 3A and 3B), followed by the decreasing of neuron-specific proteins β-tubulin III, NEFM expression (Fig 3C and 3D) and synaptic vesicle recycling as indicated by FM1-43FX fluorescence intensity (Fig 3E).

### Effects of PPAR-β knockdown on mitochondrial energy metabolism in compound 4a-induced neural progenitor cells

**Fig 4:**
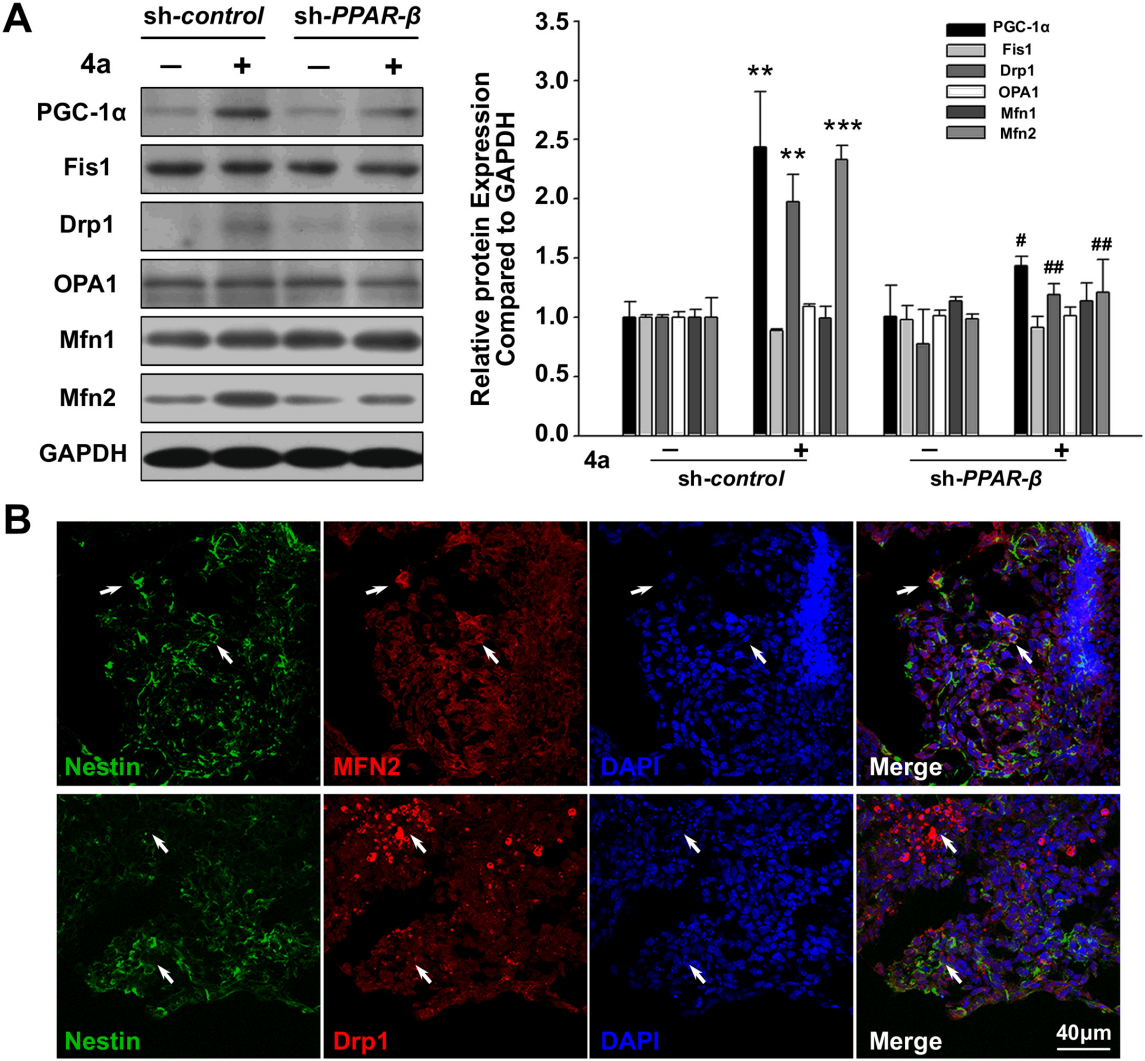
Effects of *PPAR-β* gene silencing on mitochondrial system of 4a-induced neural progenitor cells. **A:** Effects of *PPAR-β* gene silencing on mitochondrial fission and fusion function. PGC-1α is a master regulator of mitochondrial biogenesis. Fis1 and Drp1 mediate mitochondrial fission, whereas OPA1 and mitofusins (Mfn1 and Mfn2) mediate mitochondrial fusion. **B:** Double immunostaining for Drp1/Nestin and Mfn2/Nestin in **4a**-induced neural progenitor cells. Bar = 40 μm. Statistical significance was set as **P<0.01 and ***P<0.001 vs. sh-*control* group, ^#^*P*<0.05 and ^##^*P*<0.01 vs. 4a+sh-*control* group. Data represent mean ± S.D. of three independent experiments.

Compound **4a** could increase the protein expressions of mitochondrial biogenesis regulator PGC-1α, mitochondrial fission protein Drp1 and mitochondrial fusion protein Mfn2, which were abolished by sh*-PPAR-β* (Fig 4A). Three regulators of mitochondrial biogenesis, *PGC-1α*, *Nrf1* and *TFAM* mRNA expression were examined (S5 Fig). Consistently, compound **4a** could upregulate *PGC-1α*, *Nrf1* and *TFAM* mRNA expression, which were blocked by sh*-PPAR-β*, suggesting a key role of PPAR-β in mitochondrial biogenesis. We further investigated the relationship between the expressions of Drp1/Mfn2 and Nestin in neural progenitor cells. The results showed that Mfn2 was positively detected in the Nestin-positive cells. However, Drp1 was mostly detected in the Nestin-negetive cells with pyknotic nuclei (Fig 4B). The results indicated that a close relationship between PPAR-β and Mfn2 took an important role in early neuronal differentiation induced by compound **4a**, whereas Drp1 might help to induce apoptosis [29] of non-neural progenitor cells.

**Fig 5:**
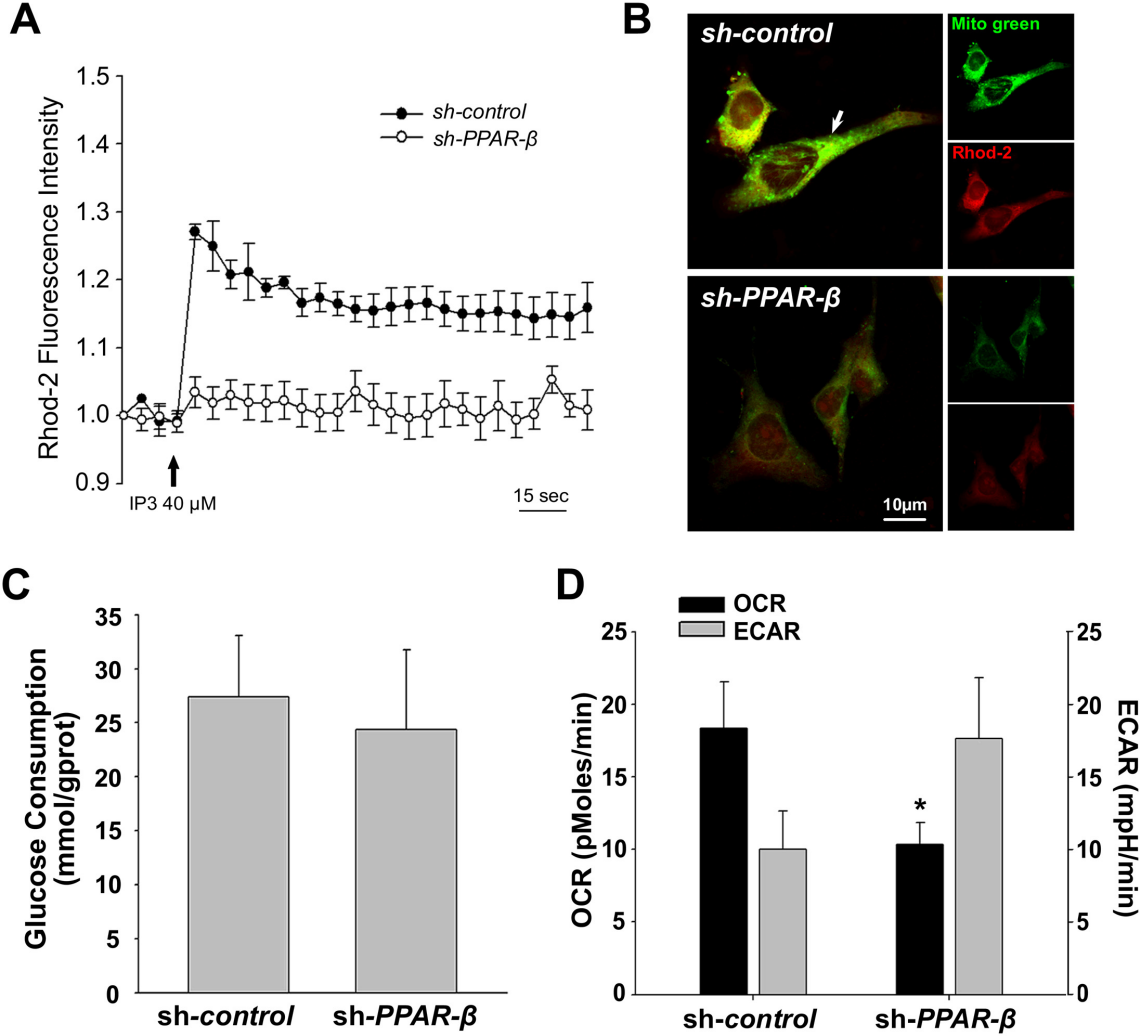
Effects of *PPAR-β* gene silencing on mitochondrial energy metabolism of 4a-induced neural progenitor cells. **A:** Mitochondrial Ca^2+^ ([Ca^2+^]_M_) transients was measured with Rhod-2AM. ES-derived neural progenitor cells were loaded with 4 μM Rhod-2AM prior to the experiment and then stimulated with 40 μM IP_3_ at the time as indicated. **B:** [Ca^2+^]_M_ concentration was revealed by co-staining of Rhod-2AM with Mito green. Bar = 10 μm. **C:** Glucose consumption was detected by a commercial glucose monitor. **D:** Basal oxygen consumption rate (OCR) and extracellular acidification rate (ECAR) in ES-derived neural progenitor cells were measured by a XF96 Extracellular Flux Analyzer, and were normalized to cell numbers. Statistical significance was set as *P<0.05 vs. sh-*control* group.

Mfn2 was reported to build a bridge between endoplasmic reticulum and mitochondria to control the efficiency of mitochondrial uptake of Ca^2+^ ions [7–9]. To investigate the effects of *PPAR-β* gene silencing on mitochondrial Ca^2+^ ([Ca^2+^]_M_), ES-derived neural progenitor cells were loaded with 4 μM Rhod-2AM prior to the experiment and then stimulated with 40 μM IP_3_ at the time as indicated. The results showed that PPAR-β knockdown affected [Ca^2+^]_M_ transients in ES-derived neural progenitor cells (Fig 5A). IP_3_-evoked [Ca^2+^]_M_ transients recorded in sh*-control* ES-derived neural progenitor cells appeared with a sharp peak, suggesting that the ability of the [Ca^2+^]_M_ buffering still kept normal. In contrast, the same ability almost disappeared in sh*-PPAR-β* group. [Ca^2+^]_M_ concentration was revealed by co-staining of Rhod-2AM with Mito green (Fig 5B). Compared to sh*-control* group, sh*-PPAR-β* decreased the fluorescence intensity of Mito green and Rhod-2, indicating that mitochondrial activity and [Ca^2+^]_M_ concentration was downregulated by sh*-PPAR-β*. The results were consistent with the previous finding that mitochondrial biogenesis and Mfn2 were affected by sh*-PPAR-β*. [Ca^2+^]_M_ could stimulate three rate-limiting enzymes in the Krebs cycle, resulting in accelerated ATP production [30]. Although glucose consumption in ES-derived neural progenitor cells was not affected by sh*-PPAR-β* (Fig 5C), energy metabolism pathway was shifted. The results in Fig 5D showed that basal oxygen consumption rate (OCR) was decreased and extracellular acidification rate (ECAR) was increased in sh*-PPAR-β* ES-derived neural progenitor cells.

## Discussion

Recent studies link changes in energy metabolism with the fate of ES cells [31, 32]. Relative shifts in metabolism from ES cells to lineage-specific differentiation place variable demands on mitochondrial biogenesis and activity for cell types with distinct energetic and biosynthetic requirements [32]. Neurons are excitable cells that need large amounts of energy to support their survival and functions [33]. By generating energy and regulating subcellular Ca^2+^, mitochondria may play essential roles in controlling fundamental processes in neuroplasticity, including neural differentiation, neurite outgrowth, and synaptic vesicle recycling [1]. Here we used flavonoid compound **4a** as a probe to dissect the involvement of mitochondrial energy metabolism in neuronal differentiation of ES cells. We further demonstrated the role of PPAR-β on the underlying mechanism, and other related molecular targets including Mfn2 and mitochondrial Ca^2+^.

It has been shown that flavonoids are key compounds for the development of a new generation of therapeutic agents that are clinically effective in treating neurodegenerative diseases [34]. Our previous work found that ICT [22] and IBA [23] had significant neurogenesis-inducing activities. Based on the core molecular scaffolds, a synthetic flavonoid library was designed and synthesized, with 2”, 3”-unsaturated alkyl groups at C-8 position as well as hydroxy or methoxy groups at different positions [24]. A cell-based phenotypic screen was undertaken to identify chemical inducers of neuronal differentiation of P19 embryonal carcinoma (EC) and mouse ES cells (data not shown). Here flavonoid compound **4a** faithfully facilitated ES cells to differentiate into neurons morphologically and functionally. Neural specific proteins were upregulated in a developmental way, providing the fundamentals for neurite outgrowth and synaptic vesicle recycling. Importantly, Nestin expression was promoted at early differentiation stage, indicating that the neurogenesis-inducing effect of compound **4a** appeared as early as neural progenitor cells formation. The FM 1-43FX fluorescence intensity in ES-derived neurons induced by **4a** was similar to that of cells treated with RA. Since synaptic vesicle recycling is a neuron-specific function, we confirmed compound **4a** could induce functional neuronal differentiation.

PPARs, which serve as lipid-activated transcription factors, play key roles in the regulation of differentiation and mitochondrial energy metabolism. It is therefore we examined the role of PPARs on the underlying mechanism of compound **4a**-induced neuronal differentiation. Expression and distribution of PPARs were revealed by double staining PPAR isotypes with neural progenitor marker Nestin. The results showed that PPAR-β had very good colocalization with Nestin-positive cells. Compound **4a** could promote PPAR-β protein expression. Treatment with PPAR-β antagonist GSK0660 abolished the neurogenesis stimulatory effect of compound **4a** with downregulation of neural specific proteins. Consistently, knockdown of PPAR-β blocked compound **4a** induced neurogenesis of ES cells, indicating the important role of PPAR-β in neuronal differentiation.

Mitochondria help neurons to meet their high energy demands of proper neuronal function [3]. Furthermore, mitochondrial biogenesis, together with mitochondrial fission and fusion, helps the transmission of energy across long distances, which is particularly essential in neurons [3]. We then analyzed the downstream events of PPAR-β involved in compound **4a**-induced neural progenitor cells. Mitochondrial biogenesis was upregulated by compound **4a** treatment, which was altered by PPAR-β knockdown, suggesting a key role of PPAR-β in mitochondrial biogenesis. Mfn2 expression is crucial in mitochondrial metabolism through the maintenance of the mitochondrial network architecture [35]. Furthermore, Mfn2 could tether endoplasmic reticulum to mitochondria, thereby increasing the efficiency of mitochondrial Ca^2+^ uptake and ATP production [7–9]. Recent research demonstrated that Mfn2 was a PPAR-β-selective target, which might play an important role in regulating myocardial energy homeostasis [36]. In accordance with these findings, present study showed that compound **4a** increased the protein expression of mitochondrial fusion protein Mfn2, which were abolished by sh*-PPAR-β*. Moreover, Mfn2 was positively detected in the Nestin-positive cells, indicating that a close relationship between PPAR-β and Mfn2 took an important role in early neuronal differentiation induced by compound **4a**.

Mitochondrial Ca^2+^ could stimulate three rate-limiting enzymes in the Krebs cycle, as a key regulator of mitochondrial ATP production in mammalian cells [30]. By determining the intermediates of energy metabolism, we found that PPAR-β knockdown affected mitochondrial Ca^2+^ buffering activity and intracellular Ca^2+^ homeostasis. Besides, PPAR-β knockdown reduced mitochondrial Ca^2+^ concentration. Consistently, energy metabolism pathway in ES-derived neural progenitor cells was shifted. OCR was decreased and ECAR was increased in sh*-PPAR-β* ES-derived neural progenitor cells, although glucose consumption was not affected. Because OCR and ECAR are indicators of mitochondrial respiration and anaerobic glycolysis pathway, the shift of mitochondrial respiration to anaerobic glycolysis might result in less ATP production and failing to fulfill the adequate level of bioenergetic capacity of neuronal differentiation.

## Conclusion

In summary, our observation demonstrated that by modulating mitochondrial energy metabolism through Mfn2 and mitochondrial Ca^2+^, PPAR-β took an important role in neuronal differentiation induced by flavonoid compound **4a**. This study provides novel insights for the role of mitochondria in the differentiation of neurons from ES cells. Further work is needed to focus on the action mechanism between compound **4a** and PPAR-β. The effect of other flavonoids on PPAR-β expression should also be investigated.

## Materials and Methods

Retinoic acid (RA), dimethylsulfoxide (DMSO), FM 1-43FX, MTT, β-mercaptoethanol (β-ME), 4,6-Diamidino-2-phenylindole (DAPI), inositol 1,4,5-triphosphate (IP3), L165041 and GSK0660 were purchased from Sigma-Aldrich (St. Louis, MO, USA). DMEM medium, fetal bovine serum (FBS), B27 supplement, neuralbasal medium was obtained from Gibco BRL (Burlington, Ontario, Canada). Non-essential amino acids (NEAA) stock solution was purchased from Hyclon (Logan, USA). Recombinant mouse leukemia inhibitory factor (LIF) was obtained from Millipore (CA, USA). Antibodies were used as follows: β-tubulin III, neuronal nuclei (NeuN), and neurofilament 160 (NEFM) were purchased from Sigma-Aldrich (St. Louis, MO, USA); synaptophysin and GAPDH were products of Cell Signaling; Nestin was from Millipore (CA, USA); PPAR-α, -β, -γ, Mfn1 and Mfn2 were ordered from Abcam; PGC-1α, Fis1, Drp1 and OPA1 were purchased from Santa Cruz Biotechnology. Secondary antibodies were purchased from Multisciences. Rhod-2AM was purchased from Dojindo Molecular Technologies. MitoTracker Green was a product of Life technologies.

### Cell culture and differentiation scheme

Mouse embryonic fibroblasts (MEF) cells were prepared as previously described [37, 38]. ICR mice (22±3 g) were obtained from the Experimental Animal Center, Zhejiang University, Hangzhou, China (License No.: scxk-Zhejiang-2004-0014). Mice were housed under 12 h light/12 h dark and 21±1°C conditions. To obtain fetuses, mice (3 female and 1 male) were housed together at 17:00. The following morning, when a copulation plug was detected, was defined as d 0 of gestation. All efforts were made to minimize the number of animals used, and their suffering. Fetuses were obtained from the mice on d 13 of gestation for the preparation of MEF cells. The MEF cells of generation three to generation five were used as feeder cells, which were treated with 1 mg/L mitomycin C for 1 h and plated at an appropriate density.

Mouse ES cells (D3 line, ATCC, CRL-1934) were routinely cultured on MEF cells in DMEM, supplemented with 10% FBS, 0.1 mM β-ME, 1×NEAA and 1×10^6^ U/L LIF. To induce neuronal differentiation, LIF was withdrawn from the medium and a typical 4−/4+ protocol was used as previously described [22, 39]. Briefly, drops (30 μL) containing about 900 ES cells were placed onto the lids of Petri dishes and the cells were cultured in hanging drops for 2 days. After EBs formed, they were transferred into agar coated Petri dishes and continued to be suspension cultured for another 2 days. On d 4, compound **4a** (final, 10^−7^ mol/L) was added into the medium and the EBs were continually suspension cultured for another 4 days. On d 8, EBs was planted onto poly-D-lysine-coated culture plates to induce the differentiation in neurobasal medium supplemented with B27, with the treatment of each compound. The culture treated with 10^−7^ mol/L RA was considered as positive control and 0.1% DMSO was set as solvent control.

To investigate whether PPAR-β agonist or antagonist would affect the neurogenesis of ES cells, EBs were treated with either L165041 (10^−5^ mol/L) or GSK0660 (10^−8^ mol/L) as previously described [10].

### Immunocytochemistry analysis

Immunostaining was performed on neuronal cultures (d 8+10) at the end of differentiation course and frozen EBs (d 8) section as previously described [22, 39]. Neuronal cultures seeded on coverslips were washed with PBS solution and then fixed for 10 min in ice-cold methanol, while frozen EBs sections were fixed in 4% paraformaldehyde for 2 h [40]. Non-specific binding sites were blocked with 10% FBS in PBS for 1 h. The cells were incubated with primary antibodies at appropriate dilutions in 0.5% triton X-100 in PBS overnight at 4°C, and then with the corresponding fluorescent secondary antibodies at a dilution of 1:200 for 2 h. Nuclear staining was performed with 2 μg/mL DAPI and the immunostaining results were visualized by microscopic examination using Leica DMI 3000B or on a confocal microscope (Fluoview FV1000, Olympus). For neuronal cultures, primary antibodies were used at the following dilutions: β-tubulin III mouse monoclonal (1:1000) and rabbit polyclonal (1:1000), NeuN rabbit polyclonal (1:1000), synaptophysin rabbit polyclonal (1:100), NEFM mouse monoclonal (1:100). While for frozen EBs section, primary antibodies were used at the following dilutions: Nestin mouse monoclonal (1:1000), PPAR-α rabbit polyclonal (1:1000), PPAR-β rabbit polyclonal (1:500), PPAR-γ rabbit polyclonal (1:500), Mfn2 rabbit polyclonal (1:500), Drp1 rabbit polyclonal (1:1000). Negative controls were performed by omitting the primary antibody. Experiments were repeated independently at least three times.

### Western blot analysis

Western blotting was performed as previously described [22, 39]. The cells were collected in RIPA buffer and lysed 30 min on ice. The lysates were centrifuged at 14000 rpm for 30 min at 4°C. An aliquot of 40 μg of the supernatant protein from each sample separated electrophoretically on 8% SDS-PAGE. Subsequently, proteins were transferred onto PVDF membranes and blocked for 1 h in 5% nonfat milk in PBS, followed by an overnight incubation at 4°C with respective antibody. The specific dilutions of the primary antibodies were as follows: Nestin (1:1000), β-tubulin III (1:1000), NEFM (1:500), synaptophysin (1:1000), PPAR-α (1:1000), PPAR-β (1:1000), PPAR-γ rabbit polyclonal (1:1000), PGC-1α (1:1000), Fis (1:1000), Drp1 (1:1000), OPA1 (1:1000), Mfn1 (1:1000), Mfn2 (1:1000) and GAPDH (1:10000). Then the membranes were incubated with HRP-conjugated secondary antibody (1:400). The proteins were visualized with an enhanced chemiluminescent substrate. The density of the products was quantitated using Quantity One version 4.6.2 software (Bio-Rad).

### FM 1-43FX fluorescent analysis

FM 1-43FX fluorescent analysis protocol was used as previously described [28, 41]. In brief, at the end of neuronal differentiation course, remove the culture medium from neuronal culture. Wash the cells for 30 s two times with 100 μl Krebs-Ringer buffer (115 mM NaCl, 5.9 mM KCl, 1.2 mM MgCl_2_, 1.2 mM NaH_2_PO_4_, 1.2 mM Na_2_SO_4_, 2.5 mM CaCl_2_, 25 mM NaHCO_3_ and 10 mM glucose) prewarmed to 37°C. Add 100 μl of prewarmed Krebs-Ringer buffer containing 100 mM potassium ions (20.9 mM NaCl, 100 mM KCl, 1.2 mM MgCl_2_, 1.2 mM NaH_2_PO_4_, 1.2 mM Na_2_SO_4_, 2.5 mM CaCl_2_, 25 mM NaHCO_3_ and 10 mM glucose) and 2 mM FM 1-43FX to the cells. Incubate the cells for 5 min at 37°C. Wash the cells for 30 s three times with 100 μl Krebs-Ringer buffer to remove excess FM 1-43FX. Add 100 μl Krebs-Ringer buffer to the cells. Measure the fluorescent intensity of the cells using DTX-880 Multimode Detector. The results across each well were then normalized by the MTT assay.

### MTT assay

MTT labeling mixture (final, 0.5 mg/mL) was added to each well and incubated for additional 4 h. The formazan precipitate was dissolved in 200 μL DMSO and measured with a DTX-880 Multimode Detector at 570 nm. Assays were performed in triplicate in three independent experiments.

### RNA silencing

Mouse PPAR-β shRNAs (sh-*PPAR-β*) with the sequences, 5’-GGAGCATCCTCACCGGC-AA-3’ and 5’-GCAGCTGGTCACTGAGCAT-3’[42] were constructed into pGPU6/GFP/Neo plasmid by GenePharm. In the PPAR-β knockdown experiments, these two plasmids were used at a 1:1 ratio. Scrambled shRNA (sh-*control*) with the sequence 5’-GTTCTCCGAA-CGTGTCACGT-3’ was used as negative control. ES cells were transfected with either sh-*PPAR-β* plasmid or sh-*control* plasmid (2 μg/mL) with lipofectamine 2000 according to the manufacturer protocol. GFP expression of ES cells was examined 48 h after transfection. To assess gene silencing, protein level of PPAR-β was determined by western blot 72 h after transfection. Viability and pluripotency of ES cells was also evaluated after transfection with sh-*PPAR-β* plasmid.

### [Ca^2+^]_M_ measurements with Rhod-2

ES-derived neural progenitor cells were loaded with 4 μmol/L Rhod-2AM [43] prior to the experiment and then stimulated with 40 μmol/L IP_3_ at the time as indicated. Single cell fluorescence was excited at 545nm and images of the emitted fluorescence obtained by a cooled CCD camera mounted on the microscope equipped with a polychromator (IX S1, Olympus). All analyses of [Ca^2+^]_M_ transients were processed at a single-cell level and expressed as the relative fluorescence intensity. [Ca^2+^]_M_ concentration was also revealed by co-staining of Rhod-2AM (4 μmol/L) with Mito green (200 nM) [44]. The images were visualized by confocal microscope (Fluoview FV1000, Olympus).

### Glucose consumption

Cell culture supernatants of a 48 h culture of ES-derived neural progenitor cells were used to quantify glucose consumption using a commercial glucose monitor (Accu-Chek, Roche). Amounts of glucose consumption were normalized to the number of cells [45].

### OCR and ECAR measurements

Oxygen consumption rate (OCR) and extra-cellular acidification rate (ECAR) were measured in ES-derived neural progenitor cells with a XF96 Extracellular Flux Analyzer (Seahorse Bioscience, Billerica, MA, USA) as previously described [46]. Cells were seeded in 12 wells of a XF 96-well cell culture microplate (Seahorse Bioscience) at a density of 10^4^ cells/well in 200 μL of DMEM and incubated for 24 h at 37°C in 5% CO_2_ atmosphere. After replacing the growth medium with 180 μL of bicarbonate-free DMEM prewarmed at 37°C, cells were preincubated for 30 min before starting the assay procedure. Data were expressed as pmol of O_2_ per minute and normalized to the number of cells.

### Statistical analysis

Data are expressed as mean values ± standard deviation (S.D.). At least three independent experiments were done. Statistical analysis was performed by Student’s t-test and one-way ANOVA. A value of *P*<0.05 was considered to be significant.

## Supporting Information

S1 Fig Protocol used to differentiate mouse ES cell into neurons. (TIFF)

S2 Fig Developmental-dependent PPARs expression during neuronal differentiation induced by compound 4a. (TIFF)

S3 Fig Effects of PPAR-β agonist L165041 or antagonist GSK0660 on compound 4a-induced neuronal differentiation. (TIF)

S4 Fig Evaluation of transfection and silencing efficiency of sh*-PPAR-β* plasmid. (TIFF)

S5 Fig Effects of *PPAR-β* gene silencing on mitochondrial biogenesis of compound 4a-induced neural precursor cells. (TIFF)

S6 Fig Change on mitochondrial energy metabolism of 4a-induced neural precursor cells. (TIFF)
