# Supplementary figures and images for: A Flavonoid Compound Promotes Neuronal Differentiation of Embryonic Stem Cells via PPAR-β Modulating Mitochondrial Energy Metabolism
Source: PLoS One. 2016 Jun 17;11(6):e0157747. doi: 10.1371/journal.pone.0157747 (PMC4912105; doi:10.1371/journal.pone.0157747)

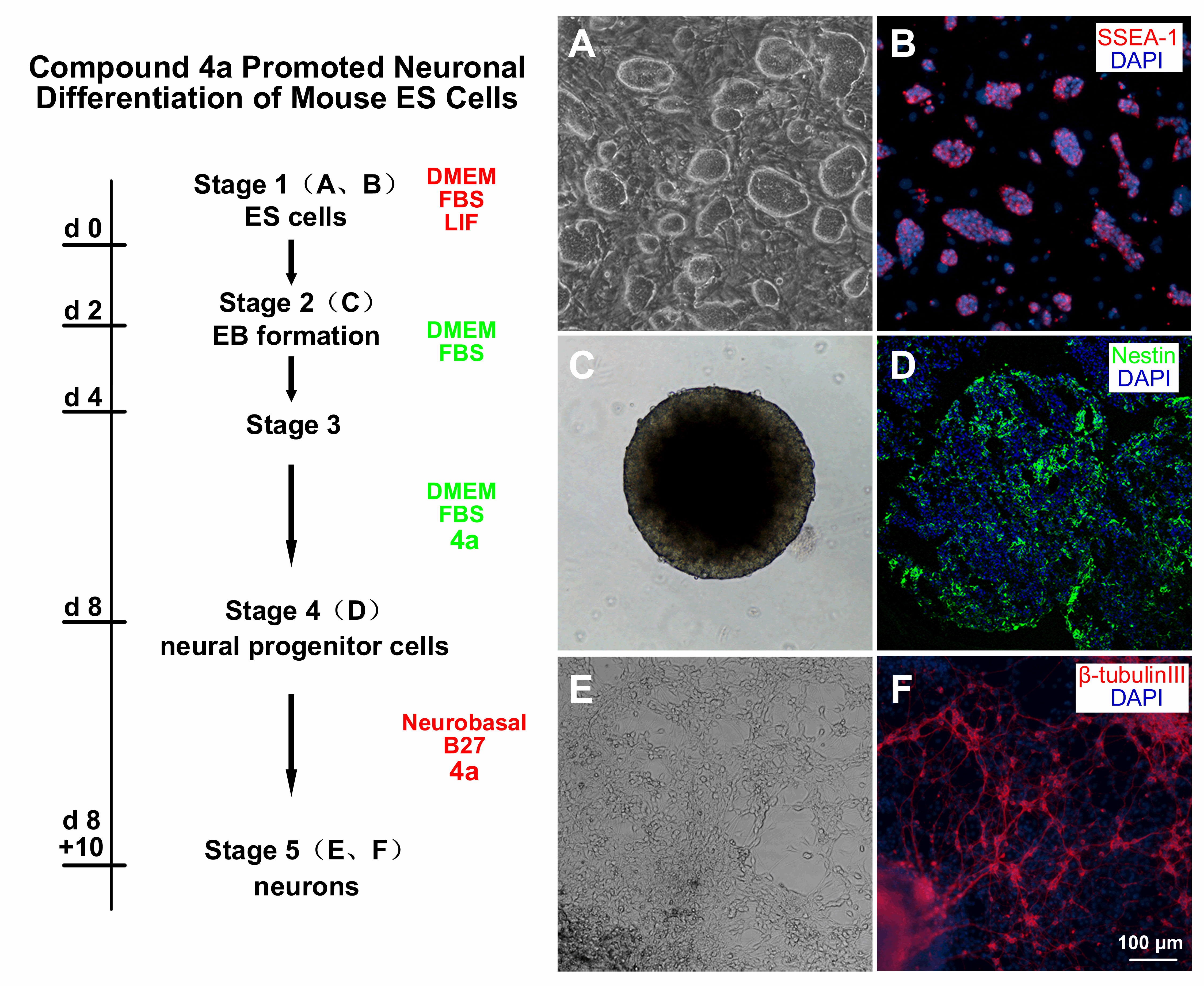

Supplement: S1 Fig — (TIFF) [file pone.0157747.s001.tiff]

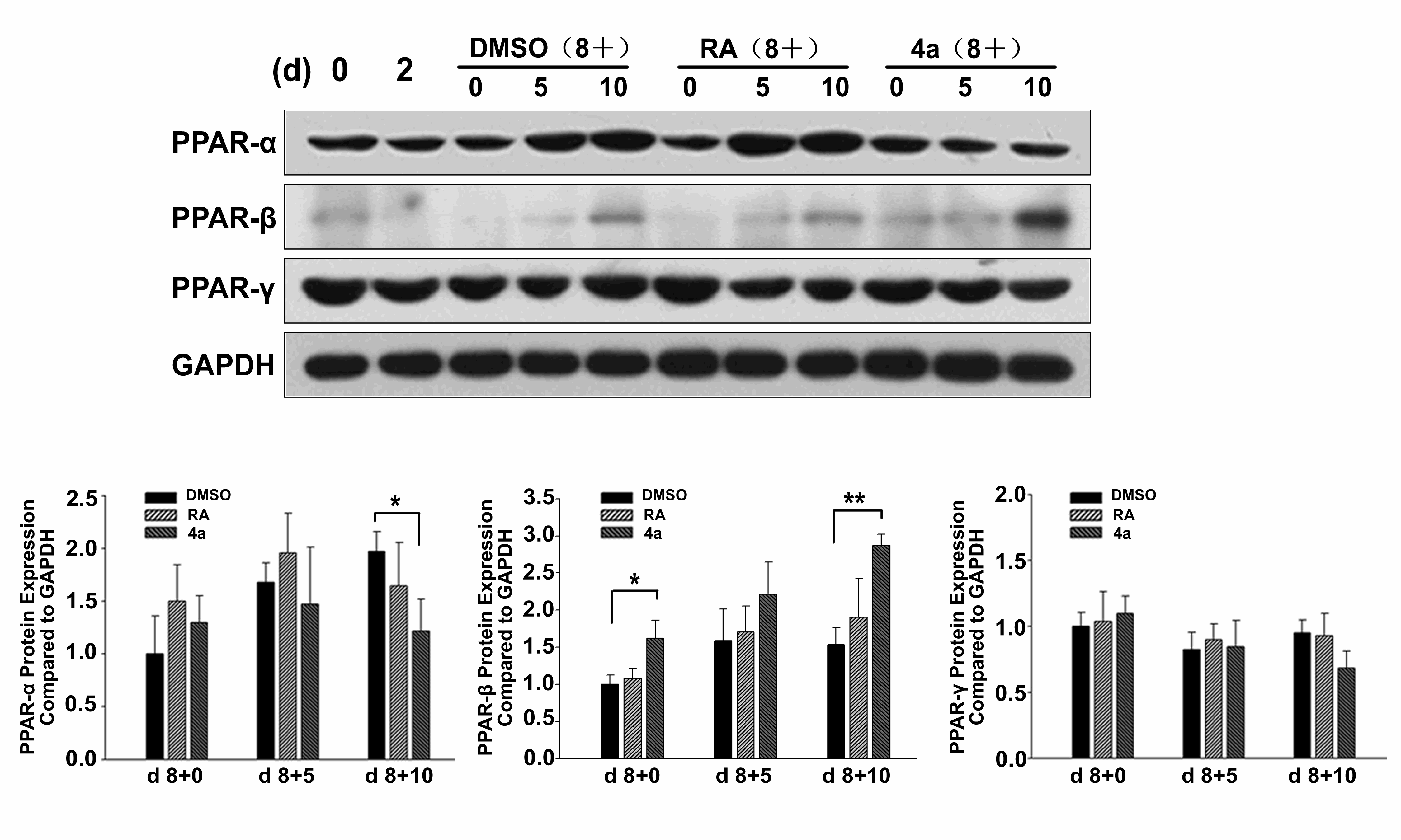

Supplement: S2 Fig — (TIFF) [file pone.0157747.s002.tiff]

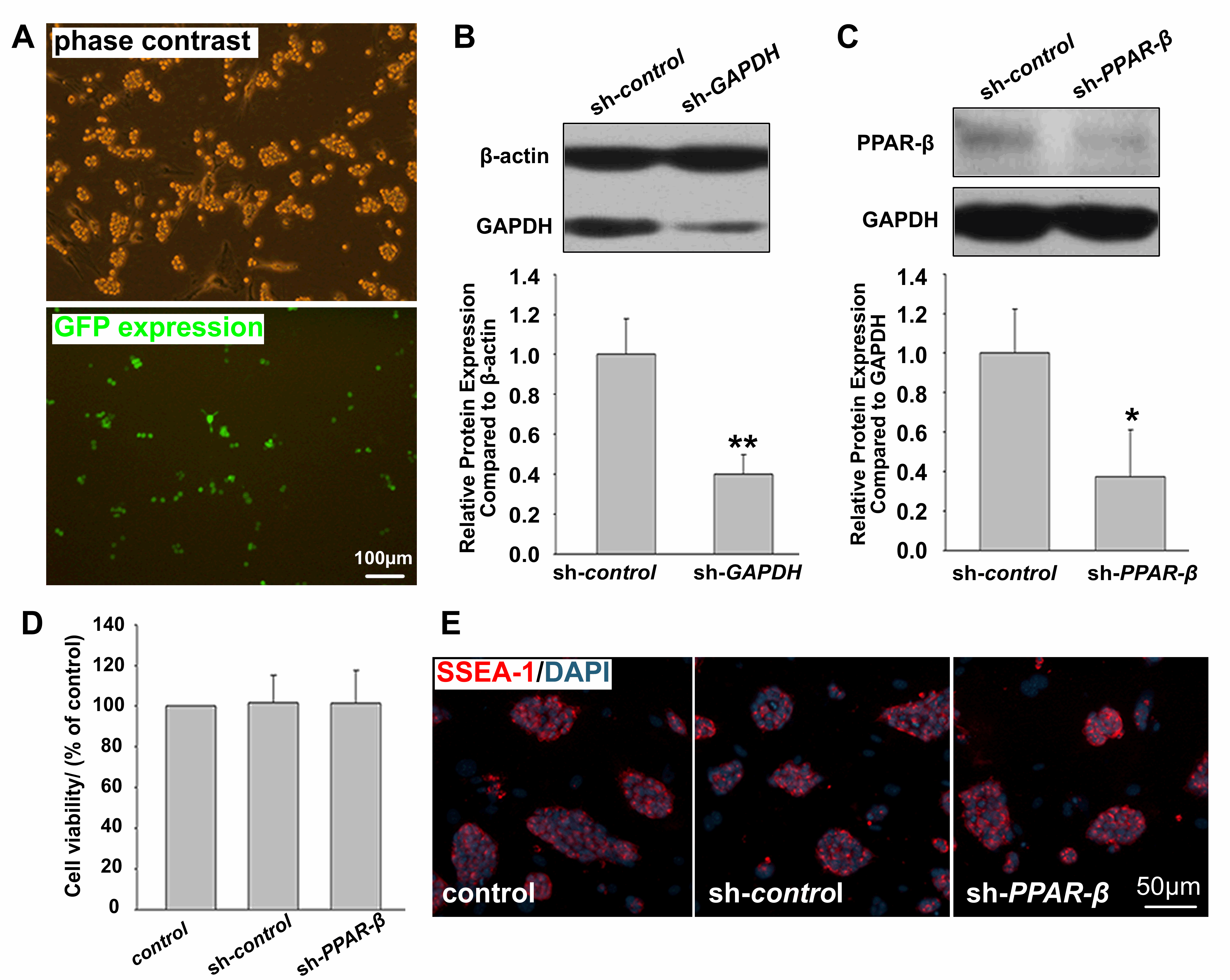

Supplement: S4 Fig — (TIFF) [file pone.0157747.s004.tiff]

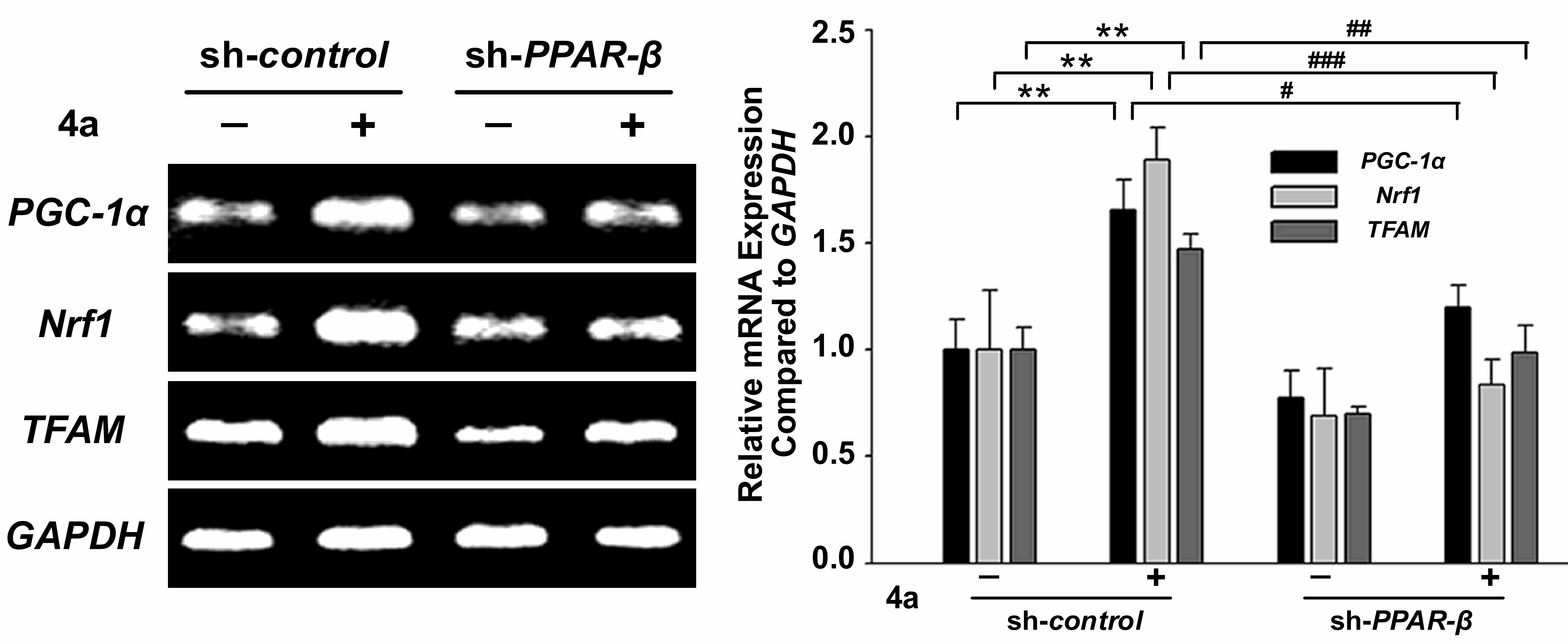

Supplement: S5 Fig — (TIFF) [file pone.0157747.s005.tiff]

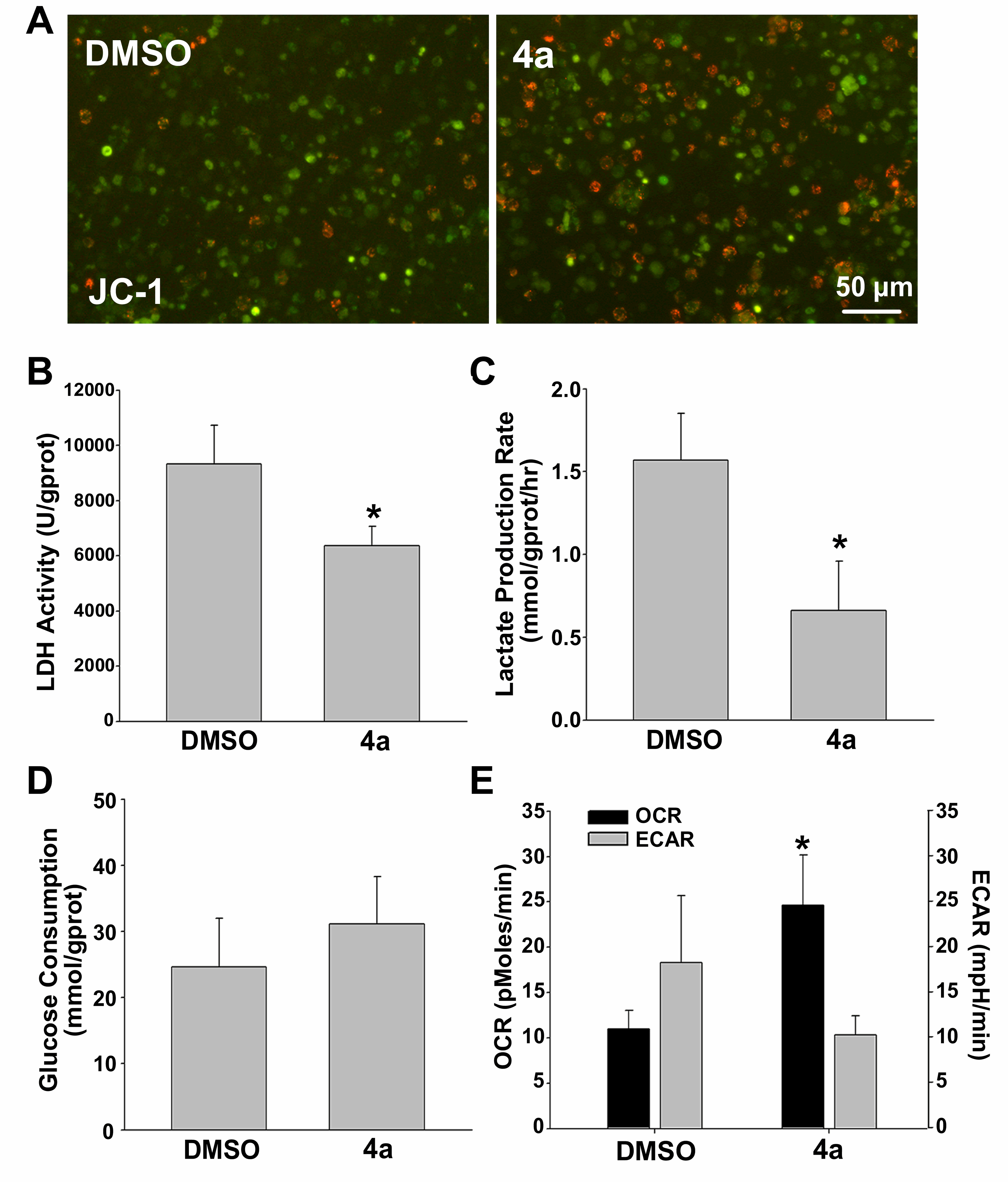

Supplement: S6 Fig — (TIFF) [file pone.0157747.s006.tiff]
